# Incidentally diagnosed anomalous left coronary artery from the pulmonary artery in a 76-year-old man undergoing preoperative evaluation: a case report and literature review

**DOI:** 10.3389/fcvm.2026.1905385

**Published:** 2026-08-24

**Authors:** Guoao Zhu, Sirui Zeng, Zihua Bao

**Affiliations:** Department of Radiology, Nanchuan Hospital of Traditional Chinese Medicine, Chongqing, China

**Keywords:** ALCAPA, Bland-White-Garland syndrome, coronary artery anomaly, coronary CT angiography, collateral circulation

## Abstract

Anomalous left coronary artery from the pulmonary artery (ALCAPA), also known as Bland-White-Garland syndrome, is a rare congenital coronary artery anomaly with an incidence of approximately 1 in 300,000 live births, accounting for 0.25%–0.5% of all congenital heart diseases. Without surgical correction, approximately 90% of affected infants die within the first year of life due to myocardial ischemia and heart failure. However, a small subset of patients with well-developed collateral circulation may survive into adulthood without marked symptoms. We report an incidentally diagnosed case of ALCAPA in a 76-year-old man who was admitted for benign prostatic hyperplasia and urinary retention and underwent cardiovascular evaluation before non-cardiac surgery. Electrocardiography showed sinus bradycardia and complete right bundle branch block, and ambulatory celectrocardiography documented frequent supraventricular premature beats and short supraventricular tachycardia runs without significant ST-T abnormalities. Echocardiography showed a dilated right coronary artery, a suspected coronary fistula, mild chamber enlargement, preserved left ventricular systolic function, and no typical resting segmental wall-motion abnormality. Coronary CT angiography (CTA), performed after cardiology consultation, demonstrated the left main coronary artery arising from the pulmonary trunk, a markedly dilated and tortuous right coronary artery, extensive collateral circulation among the right coronary artery, left coronary system, and bronchial arteries, and a high coronary artery calcium score of 941.74 Agatston units. Cardiac enzymes were not elevated, but formal stress testing and cardiac magnetic resonance imaging were not performed during the index admission. The management decision was therefore framed as individualized conservative follow-up rather than evidence that conservative treatment is generally safe in adult ALCAPA. This case illustrates the diagnostic value of CTA for defining coronary anatomy in late adult ALCAPA, the need for functional ischemia and scar assessment when feasible, and the therapeutic tension between guideline-based surgical recommendations and individualized decision-making in elderly patients with preserved resting ventricular function.

## 1 Introduction

Anomalous origin of the left coronary artery from the pulmonary artery (ALCAPA) is a rare congenital coronary anomaly first described by Bland, White, and Garland in 1933 (1). With an incidence of approximately 1:300,000 live births, it accounts for 0.25%–0.5% of all congenital heart diseases (2). The hemodynamic consequences of ALCAPA depend on the balance between myocardial oxygen demand and the adequacy of collateral circulation. In the classic infantile type, the left coronary artery (LCA) perfuses the left ventricle with deoxygenated blood at low pressure shortly after birth as pulmonary vascular resistance falls, leading to profound myocardial ischemia, left ventricular dysfunction, and mitral regurgitation. Without timely surgical intervention, mortality approaches 90% within the first year of life (1, 3).

However, approximately 10%–15% of ALCAPA patients survive to adulthood without surgical repair, representing the so-called adult type (4). In these patients, gradual development of extensive intercoronary collateral networks from the right coronary artery (RCA) to the LCA territory provides partial compensation for myocardial perfusion. The RCA is typically markedly dilated and tortuous, and collateral circulation may involve not only intercoronary connections but also bronchial and mediastinal collateral vessels (5, 6). Despite this compensatory mechanism, adult ALCAPA patients remain at risk for long-term complications including myocardial ischemia, arrhythmia, left ventricular dysfunction, and sudden cardiac death (7, 8).

With the widespread use of non-invasive cardiac imaging, particularly coronary CT angiography (CTA), an increasing number of adult ALCAPA cases are being identified incidentally (9, 10). CTA provides high-resolution anatomical delineation of the anomalous origin, coronary course, collateral vessel morphology, and associated coronary atherosclerosis; however, CTA alone does not replace functional testing for inducible ischemia, myocardial viability, scar, shunt burden, or coronary steal physiology. We report a case of incidentally diagnosed ALCAPA in a 76-year-old man and discuss the diagnostic pathway, late-adult novelty, limitations of available risk stratification, and management considerations.

## 2 Case report

### 2.1 Clinical presentation and diagnostic sequence

A 76-year-old man was admitted to the Department of Surgery on December 4, 2025 because of progressive dysuria for 4 years with acute worsening for 8 h. The admitting diagnosis was benign prostatic hyperplasia with urinary retention and chronic bronchitis. He had no known history of coronary artery disease, hypertension, diabetes mellitus, or congenital heart disease. Retrospective history was partly inconsistent across clinical notes: the pre-anesthesia consultation documented no usual palpitations, chest pain, or syncope and noted that the patient performed light physical activity at home, whereas the cardiology consultation recorded a family report of an unspecified vascular anomaly diagnosed 4 years earlier and intermittent chest tightness, palpitations, and shortness of breath. We therefore describe the ALCAPA as incidentally detected during preoperative cardiovascular evaluation rather than as a proven lifelong asymptomatic condition.

As part of the preoperative assessment, a 12-lead electrocardiogram on December 4, 2025 showed sinus bradycardia and complete right bundle branch block. Transthoracic echocardiography performed the same day showed mild left atrial enlargement, dilatation of the right coronary artery (9.6 mm) and left coronary artery (6.7 mm), suspected right coronary artery fistula into the right ventricle, interventricular septal thickening, aortic valve calcification, mild mitral, tricuspid, and aortic regurgitation, preserved left ventricular systolic function (ejection fraction 70%), grade I diastolic dysfunction, and a small pericardial effusion. No typical resting segmental wall-motion abnormality was seen.

Twenty-four-hour ambulatory electrocardiography from December 5 to December 6, 2025 revealed 94,761 total beats, a mean heart rate of 68 bpm, a minimum heart rate of 50 bpm, and a maximum sinus heart rate of 103 bpm. There were 5,761 supraventricular premature beats (5.98%), including 241 couplets, 68 bigeminal episodes, 94 trigeminal episodes, and 53 short supraventricular tachycardia runs, with the longest run lasting 10 beats. Forty ventricular premature beats were recorded. No significant ST-segment or T-wave abnormalities, atrial fibrillation, atrial flutter, ventricular tachycardia, or pauses ≥1.5 s were detected. Heart rate variability was mildly reduced, and complete right bundle branch block was again noted.

On December 7, 2025, cardiology consultation was requested for perioperative cardiac risk assessment and medication adjustment. The consultant noted the abnormal ECG/Holter findings, echocardiographic suspicion of a coronary fistula or coronary abnormality, and an elevated N-terminal pro-B-type natriuretic peptide level of 1,079 pg/mL, and recommended cardiac enzyme testing and coronary CTA to further assess the coronary anatomy and perioperative risk. Cardiac biomarkers showed no evidence of acute myocardial injury (troponin I 0.006 ng/mL; creatine kinase-MB 1.62 ng/mL). Anesthesia consultation documented no absolute contraindication to spinal anesthesia but advised explicit perioperative risk communication because of the possibility of malignant arrhythmia, acute heart failure, myocardial ischemia, cardiac arrest, or cerebrovascular events.

**Figure 1:**
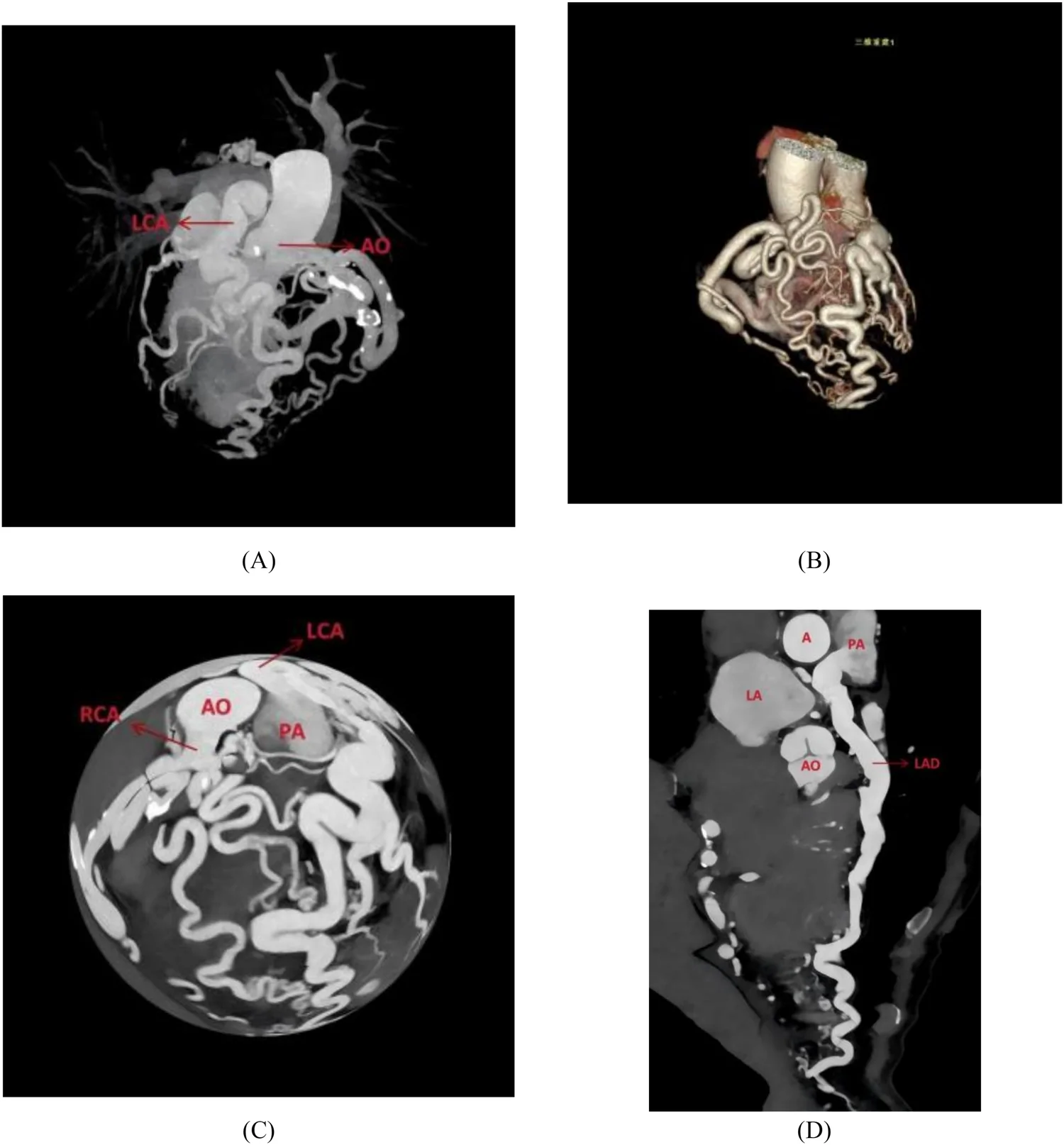
Coronary CTA in a 76-year-old man with ALCAPA. **(A)** Maximum intensity projection image showing anomalous origin of the left main coronary artery (LMCA, arrow) from the pulmonary artery (PA), providing direct anatomical evidence of ALCAPA. **(B)** Volume-rendered reconstruction showing a markedly dilated and tortuous right coronary artery (RCA) and extensive epicardial collateral vessels. **(C)** Panoramic coronary reconstruction showing collateral filling of the left coronary system, including the left anterior descending and left circumflex arteries. **(D)** Curved planar reformation of the LMCA confirming its origin from the pulmonary artery and delineating the proximal anomalous vessel. The figure demonstrates anatomical collateralization but does not by itself establish absence of inducible ischemia, myocardial scar, or coronary steal physiology.

Coronary CTA was performed on December 7, 2025 using a 64-slice multidetector CT scanner. Imaging revealed the following key findings:

1. Coronary artery atherosclerosis: Calcified and non-calcified plaques were observed in the proximal, mid, and distal segments of the RCA, causing mild luminal stenosis. Calcified plaques were also present in the proximal acute marginal and left ventricular posterior branches, with mild stenosis. The coronary artery calcium score was 941.74 Agatston units.
2. Congenital anatomical anomaly: The left main coronary artery (LMCA) originated anomalously from the pulmonary trunk (Figures 1A–D). The LMCA, left anterior descending artery, and left circumflex artery were dilated and tortuous.
3. Extensive collateral circulation: A rich network of collateral vessels was identified among the RCA territory, the LCA territory, and the bronchial arteries, providing collateral supply to the left coronary system (Figure 1).
4. Cardiac structural changes: The left atrium and left ventricle were enlarged, the left ventricular wall was thickened, and multiple small intramyocardial vascular channels were visible.

Based on these findings, adult-type ALCAPA with extensive collateral circulation was diagnosed. Provocative stress testing, nuclear perfusion imaging, stress echocardiography, invasive hemodynamic assessment, and cardiac magnetic resonance imaging with late gadolinium enhancement were not performed during the index admission. Thus, inducible ischemia, myocardial scar/fibrosis, viability, shunt magnitude, and coronary steal physiology were not objectively assessed.

The chronological diagnostic pathway is summarized in Table 1, emphasizing that CTA was obtained after abnormal preoperative cardiovascular findings and cardiology consultation rather than as routine screening.

**Table 1:** Chronological clinical timeline.

Date	Event
December 4, 2025	Admission for progressive dysuria and urinary retention due to benign prostatic hyperplasia. Preoperative ECG showed sinus bradycardia and complete right bundle branch block. Echocardiography showed preserved LVEF, dilated coronary arteries, suspected right coronary artery fistula, and no typical resting segmental wall-motion abnormality.
December 5–6, 2025	Ambulatory ECG showed frequent supraventricular premature beats and short supraventricular tachycardia runs, rare ventricular premature beats, no significant ST-T abnormalities, and no long pauses.
December 7, 2025	Cardiology consultation recommended cardiac enzymes and coronary CTA because of abnormal ECG/Holter findings and suspected coronary fistula/coronary anomaly. Cardiac biomarkers were not elevated. Coronary CTA demonstrated ALCAPA, extensive RCA-LCA-bronchial collateralization, mild RCA atherosclerotic stenosis, and a calcium score of 941.74 Agatston units.
December 8, 2025	The patient underwent transurethral vaporization/resection for prostatic hyperplasia under spinal anesthesia.
December 9–14, 2025	In-hospital postoperative course was clinically stable. The patient had no documented acute cardiovascular event, heart failure episode, myocardial infarction, or malignant arrhythmia.
December 14, 2025	Discharged after urological recovery with outpatient follow-up advice. No dedicated post-discharge cardiology follow-up, repeat Holter, stress imaging, CMR, or repeat coronary imaging was available at the time of manuscript revision.

### 2.2 Management, in-hospital outcome, and follow-up status

Current adult congenital heart disease guidance recommends surgical repair for adults with anomalous coronary artery arising from the pulmonary artery (11). In the present patient, the immediate clinical context was perioperative risk assessment before non-cardiac surgery rather than planned ALCAPA repair. Given the advanced age, preserved resting LVEF, absence of acute myocardial injury, lack of significant ST-T abnormalities on ambulatory ECG, extensive collateralization on CTA, and the patient's clinical stability during the index admission, the clinical team planned individualized conservative follow-up rather than immediate surgical intervention. This decision should not be interpreted as guideline-level evidence in favor of conservative management; it reflects individualized risk-benefit judgment in an elderly patient with incomplete functional risk stratification.

The urological operation was performed on December 8, 2025 under spinal anesthesia. During the remaining hospital stay, the patient remained clinically stable and no acute cardiovascular event, heart failure episode, myocardial infarction, or malignant arrhythmia was documented. He was discharged on December 14, 2025 with outpatient follow-up advice. Dedicated post-discharge cardiology follow-up, repeat ECG/Holter monitoring, stress testing, CMR, repeat CTA, and long-term cardiovascular outcomes were not available at the time of this revision.

## 3 Discussion

This case has three main clinical contributions. First, it adds a very late adult male case of incidentally detected ALCAPA to the small body of elderly ALCAPA literature. Second, it illustrates a realistic diagnostic pathway in which routine preoperative testing, echocardiographic suspicion of a coronary abnormality, and cardiology consultation led to coronary CTA. Third, it highlights the uncertainty of conservative management when anatomical compensation appears robust but objective functional ischemia and scar assessment are unavailable.

The natural history of ALCAPA is highly variable and depends critically on collateral circulation. In fetal life, both the pulmonary artery and aorta have similar oxygen saturation and pressure, so myocardial perfusion is not compromised. After birth, as pulmonary vascular resistance decreases, the anomalous LCA becomes perfused with deoxygenated blood at low pressure, creating a steal phenomenon from the LCA territory into the pulmonary artery (12). In patients with insufficient collaterals, this leads to the classic infantile presentation with severe myocardial ischemia and high mortality. In contrast, patients with well-developed collaterals may remain minimally symptomatic or be diagnosed incidentally in adulthood (4, 13).

The left-to-left or right-to-left intercoronary collateral circulation in ALCAPA typically involves connections between the conus branch of the RCA and the LCA, septal perforators, and apical collateral networks (5). When collaterals are sufficiently developed, blood may flow from the RCA through collaterals into the LCA system and then into the pulmonary artery, producing coronary steal physiology (12, 14). This flow pattern may be suggested by Doppler echocardiography or by delayed opacification of the LCA from collateral filling on CTA or conventional angiography, a sign sometimes described as ghost LCA or retrograde filling (15). In our patient, CTA clearly demonstrated the anomalous origin and extensive collateral anatomy, but the direction and physiological magnitude of flow were not quantified.

Several late-adult ALCAPA cases have been reported. Yau et al. (4) reviewed 151 adult ALCAPA cases and included patients up to 83 years of age. De Stefano et al. (16) described a 76-year-old woman with late adult-type ALCAPA, Vizzuso et al. (17) reported a 70-year-old man, and Cambronero-Cortinas et al. (13) described a 65-year-old asymptomatic woman. Accordingly, the novelty of the present case is not age alone. Its contribution lies in the combination of advanced age, male sex, incidental perioperative discovery, clearly demonstrated RCA-LCA-bronchial collateralization, preserved resting LVEF, and explicit documentation of the limits of available functional risk stratification.

To make this comparison explicit, Table 2 summarizes selected late-adult ALCAPA reports that frame the novelty of the present case without relying on age alone.

**Table 2:** Selected late-adult ALCAPA reports relevant to the novelty of the present case.

Report	Age (years)	Sex	Clinical presentation	Main diagnostic modality	Ischemia/scar testing	LV function/arrhythmia	Management and follow-up
Yau et al. (4)	Up to 83	Both	Adult ALCAPA review of 151 cases	Multiple modalities	Variable across cases	Variable across cases	Review emphasizing heterogeneous adult presentations and management.
Cambronero-Cortinas et al. (13)	65	Female	Very late asymptomatic presentation	Multimodality imaging	Not reported	Not reported	Case report; management details should be interpreted in its individual clinical context.
Vizzuso et al. (17)	70	Male	Late adult presentation	Coronary angiography/CTA	Not reported	Not reported	Case report of a 70-year-old male patient.
De Stefano et al. (16)	76	Female	Late adult-type ALCAPA	Imaging-based diagnosis	Not reported	Not reported	Case report of a 76-year-old female patient.
Present case	76	Male	Incidentally detected during preoperative evaluation; no typical angina or syncope documented during admission	CTA after echocardiographic suspicion of coronary abnormality	Stress testing and CMR not performed	LVEF 70%; RBBB; frequent supraventricular premature beats; no Holter ST-T change	Individualized conservative follow-up plan; stable in-hospital course; no dedicated post-discharge cardiology follow-up available.

ALCAPA, anomalous left coronary artery from the pulmonary artery; CMR, cardiac magnetic resonance; CTA, computed tomography angiography; LV, left ventricular; LVEF, left ventricular ejection fraction; RBBB, right bundle branch block.

CTA was central to diagnosis in this case. It confirmed the left main coronary origin from the pulmonary trunk, depicted a markedly dilated and tortuous RCA, showed collateralization involving the RCA, LCA, and bronchial arteries, and simultaneously identified substantial coronary calcification with only mild luminal stenosis in the RCA territory. The high calcium score indicates a large burden of calcified coronary atherosclerosis and should be managed as an independent cardiovascular risk marker in addition to the congenital anomaly. However, in ALCAPA, anatomical imaging should ideally be complemented by functional assessment when feasible, including exercise or pharmacologic stress testing, stress echocardiography, nuclear perfusion imaging, CMR perfusion and late gadolinium enhancement, invasive hemodynamic assessment, or shunt evaluation. Because these tests were not performed in our patient, silent ischemia, myocardial scar/fibrosis, viability, and steal physiology could not be excluded.

ECG findings in adult ALCAPA are non-specific but may include evidence of prior myocardial infarction, left ventricular hypertrophy, conduction abnormalities, arrhythmias, and ST-T changes (18). Our patient had complete right bundle branch block and frequent supraventricular ectopy with short supraventricular tachycardia runs, while Holter monitoring showed no significant ST-T abnormalities and no malignant ventricular arrhythmia. These rhythm findings may be age-related, but in the setting of ALCAPA they also raise the possibility of subclinical ischemia or myocardial fibrosis. Without CMR or stress imaging, the mechanism cannot be determined.

Management of adult ALCAPA is challenging. Surgical correction, typically involving direct reimplantation of the LCA into the aorta or coronary artery bypass grafting, is standard for pediatric ALCAPA and is recommended for adults with anomalous coronary artery from the pulmonary artery in adult congenital heart disease guidelines (1, 11, 19). Surgical adult series are informative but not directly transferable to every elderly patient. For example, Le Berre et al. (20) reported adult ALCAPA patients presenting in adulthood, most of whom underwent surgery; however, that cohort was symptomatic and does not prove that conservative management is safe for an incidentally diagnosed elderly patient. In our case, conservative follow-up was chosen in the context of advanced age, preserved resting systolic function, extensive collateralization, no acute myocardial injury, and perioperative clinical stability. The absence of stress testing, CMR, invasive hemodynamics, and long-term follow-up is a major limitation and prevents any broad conclusion about conservative management.

## 4 Conclusion

We report an incidentally diagnosed case of ALCAPA in a 76-year-old man undergoing preoperative evaluation for non-cardiac surgery. CTA provided definitive anatomical diagnosis and demonstrated extensive RCA-LCA-bronchial collateralization, while echocardiography showed preserved resting LVEF and Holter monitoring showed no significant ST-T abnormality. Nevertheless, the absence of stress testing, CMR, shunt/hemodynamic assessment, and dedicated long-term cardiology follow-up means that silent ischemia, myocardial fibrosis, coronary steal physiology, and late risk could not be excluded. This case supports the role of CTA in anatomical diagnosis of late adult ALCAPA and underscores that management in elderly patients should be individualized, explicitly balancing guideline-based surgical recommendations, anatomical findings, functional risk assessment when available, comorbidity, operative risk, and patient preference.

## Data Availability

The original contributions presented in this study are included in the article and supplementary material. Further inquiries can be directed to the corresponding author.
