# Supplementary material for: Incidentally diagnosed anomalous left coronary artery from the pulmonary artery in a 76-year-old man undergoing preoperative evaluation: a case report and literature review
Source: Front Cardiovasc Med. 2026 Aug 24;13:1905385. doi: 10.3389/fcvm.2026.1905385 (PMC13547925; doi:10.3389/fcvm.2026.1905385)
Supplement: Supplementary file 1 [file Datasheet1.pdf]

Supplementary Material

Incidentally diagnosed anomalous left coronary artery from the pulmonary artery (ALCAPA) in a 76-year-old man undergoing preoperative evaluation

De-identified supplementary file prepared for manuscript revision

Supplementary Note

This supplementary file contains de-identified reporting material prepared for manuscript submission. It includes a completed CARE checklist, a concise clinical timeline, and an index of the source materials used to revise the case report. Raw hospital records, ECG images, DICOM files, and consultation PDFs were not included in this submission package because the local source files contain direct patient identifiers. If the journal requests original source documentation or imaging data, those files should be de-identified by the hospital before upload.

Supplementary Table S1. CARE Checklist

Item	CARE topic	Where addressed in revised manuscript	Notes
1	Title	Title page	Identifies the case report, diagnosis, and clinical context of preoperative incidental detection.
2	Keywords	Keywords	Includes ALCAPA, Bland-White-Garland syndrome, coronary artery anomaly, CTA, elderly, case report, and collateral circulation.
3	Abstract	Abstract	Summarizes presentation, diagnostic pathway, CTA diagnosis, available risk data, missing stress/CMR assessment, and management interpretation.
4	Introduction	Introduction	Provides disease background, adult-type ALCAPA context, and rationale for reporting a very late adult case.
5	Patient information	Case report: Clinical presentation and diagnostic sequence	Describes age, sex, presenting urological complaint, relevant negative history, and inconsistent retrospective symptom documentation.
6	Clinical findings	Case report	Describes ECG, Holter, echocardiography, cardiac biomarkers, and clinical examination/consultation findings available from the index admission.
7	Timeline	Manuscript Table 1 and Supplementary Table S2	Provides date-based chronology from admission to discharge.
8	Diagnostic assessment	Case report and Figure 1	Describes coronary CTA findings, anomalous LMCA origin, collateral circulation, coronary calcium score, and the absence of formal stress/CMR/hemodynamic testing.

Item	CARE topic	Where addressed in revised manuscript	Notes
9	Therapeutic intervention	Management, in-hospital outcome, and follow-up status	Describes individualized conservative follow-up planning and the non-cardiac operation performed under spinal anesthesia.
10	Follow-up and outcomes	Management, in-hospital outcome, and follow-up status	Reports stable in-hospital postoperative course and discharge status; states that dedicated post-discharge cardiology follow-up was not available at revision.
11	Discussion	Discussion	Discusses late-adult ALCAPA, collateral circulation, CTA role, calcium score, ECG/Holter interpretation, guideline-based surgical context, and limitations.
12	Patient perspective	Not available	A formal patient-perspective statement was not available in the supplied materials.
13	Informed consent	Ethics Statement	The manuscript states that written informed consent was obtained for publication of the case report and accompanying images.

Supplementary Table S2. De-identified Clinical Timeline

Date	Clinical event
December 4, 2025	Admission for progressive dysuria and urinary retention attributed to benign prostatic hyperplasia. Preoperative ECG showed sinus bradycardia and complete right bundle branch block. Echocardiography showed preserved LVEF, dilated coronary arteries, suspected right coronary artery fistula, and no typical resting segmental wall-motion abnormality.
December 5–6, 2025	Twenty-four-hour ambulatory ECG showed frequent supraventricular premature beats and short supraventricular tachycardia runs, rare ventricular premature beats, no significant ST-T abnormality, no atrial fibrillation/flutter, and no long pause.
December 7, 2025	Cardiology consultation recommended cardiac enzymes and coronary CTA because of abnormal ECG/Holter findings and suspected coronary fistula or coronary anomaly. Cardiac biomarkers were not elevated. CTA demonstrated ALCAPA, extensive RCA-LCA-bronchial collateralization, mild RCA atherosclerotic stenosis, and a calcium score of 941.74 Agatston units.
December 8, 2025	The patient underwent transurethral surgery for benign prostatic hyperplasia under spinal anesthesia.
December 9–14, 2025	Postoperative in-hospital course was clinically stable, with no documented acute cardiovascular event, heart failure episode, myocardial infarction, or malignant arrhythmia.
December 14, 2025	Discharged after urological recovery with outpatient follow-up advice. No dedicated post-discharge cardiology follow-up, repeat Holter, stress imaging, CMR, or repeat coronary imaging was available at manuscript revision.

Supplementary Table S3. De-identified Source Material Index

Source material category	Used for revision	Submission handling
12-lead ECG image/report	Preoperative ECG abnormality and complete right bundle branch block.	Not included in this submission package because the source image contains identifiers. De-identification is required before journal upload if requested.
24-hour ambulatory ECG report	Heart rate range, supraventricular premature beats, short SVT runs, rare ventricular premature beats, absence of significant ST-T abnormality, no long pauses.	Not included directly because the report contains identifiers; summarized in manuscript and timeline.
Transthoracic echocardiography report	Preserved LVEF, chamber size, suspected coronary fistula, dilated coronary arteries, valvular findings, and absence of typical resting segmental wall-motion abnormality.	Not included directly because the report contains identifiers; summarized in manuscript and timeline.
Cardiology consultation record	Rationale for coronary CTA and perioperative cardiac risk assessment.	Not included directly because the record contains identifiers; summarized in manuscript.
Anesthesia consultation record	Perioperative risk communication and absence of absolute anesthesia contraindication.	Not included directly because the record contains identifiers; summarized in manuscript.
Laboratory report	Normal troponin I and CK-MB; elevated NT-proBNP; renal function context.	Not included directly because the report contains identifiers; summarized in manuscript.
Coronary CTA report and images	ALCAPA diagnosis, anomalous LMCA origin, RCA-LCA-bronchial collaterals, RCA atherosclerosis, calcium score, and figure panels.	De-identified figure panels are included in the revised manuscript. Raw DICOM files are not included because they may contain metadata identifiers.
Discharge summary and course records	In-hospital outcome, discharge date/status, and lack of documented post-discharge cardiology follow-up in supplied materials.	Not included directly because the records contain identifiers; summarized in manuscript and timeline.

Limitations Relevant to Supplementary Material

The supplied source materials did not include formal exercise or pharmacological stress testing, nuclear perfusion imaging, stress echocardiography, cardiac MRI with late gadolinium enhancement, invasive hemodynamic assessment, shunt quantification, or dedicated post-discharge cardiology follow-up. These limitations are explicitly acknowledged in the revised manuscript.
